# The role of histamine in opening blood-tumor barrier

**DOI:** 10.18632/oncotarget.8896

**Published:** 2016-04-21

**Authors:** Zeng Wang, Xin-jun Cai, Jing Qin, Fa-Jun Xie, Na Han, Hong-yang Lu

**Affiliations:** ^1^ Zhejiang Key Laboratory of Diagnosis & Treatment Technology on Thoracic Oncology (Lung and Esophagus), Zhejiang Cancer Hospital, Hangzhou, 310022, P.R. China; ^2^ Department of Pharmacy, Zhejiang Cancer Hospital, Hangzhou, 310022, P.R. China; ^3^ Department of Pharmacy, Integrated Chinese and Western Medicine Hospital of Zhejiang Province, Hangzhou, 310003, P.R. China; ^4^ Department of Thoracic Medical Oncology, Zhejiang Cancer Hospital, Hangzhou, 310022, P.R. China

**Keywords:** histamine, H2 receptor, tight junctions-associated proteins, blood-tumor barrier

## Abstract

Blood-tumor barrier (BTB) reduce the permeability for drugs into tumor tissues. We found that histamine might serve as an essential regulator of BTB function. Further, we aim to determine the role of H2 receptor expression in BTB permeability, and elucidate the underlying mechanisms thereof. Transmission electron microscopy showed that histamine disrupted the integrity of tight junctions (TJ) and increased the number of pinosomes in the cytoplasm. Horseradish peroxidase (HRP) and trans-endothelial resistance detection (TEER) assays revealed that histamine could open BTB and this action was inhibited by cimetidine. Western blot and immunofluorescence assays showed that histamine decreased the expression of tight junction proteins zonula occluden-1(ZO-1), occludin, and claudin-5. Further, quantitative RT-PCR assay showed that the expression of H2 receptor could represent and predicted histamine-induced BTB permeability. In conclusion, histamine opened BTB by down-regulating the TJ-associated proteins. The levels of H2 receptor expression was correlated with the histamine-induced BTB permeability.

## INTRODUCTION

Brain tumor capillaries contain a blood-tumor barrier (BTB) that limits the delivery of anti-tumor agents to tumor and the surrounding brain tissue [1, 2]. Drug penetration across the BTB is mediated by the opening of tight junctions (TJ) in the paracellular route and increased transcellular transportation [3]. TJ is located across consecutive endothelial cells of brain capillaries which are composed of transmembrane proteins including claudins, occludins, zonula occludin proteins and junctional adhesion molecules. Studies have demonstrated that zonula occluden-1(ZO-1), occludin and claudin-5 proteins can be used as characteristic markers of TJ, down-regulation of which opens the TJ and leads to increased permeability of BTB [4, 5]. Caveolae are saccate structures that contribute to surface specificity of cytomembrane and represent the first definitive structure that mediate endocytosis of endothelial cells. Caveolin is consisting of caveolin-1, caveolin-2 and caveolin-3 proteins and is mainly mediated by the transcellular transportation of micro and macromolecules after endocytosis. Caveolin-1 plays a key role in maintaining the appearance, structure and function of caveolae [6]. Studies show that vasoactive substances such as bradykinin (BK) and histamine increase the permeability of BTB [7]. In 1994, Nomura et al. found that low dose of BK selectively open BTB without affecting the permeability of normal brain tissue [8]. Researchers at the China Medical University demonstrated that opening of BTB in BK was associated with the expression levels of TJ proteins ZO-1, occludin, claudin-5 and cytoskeletal protein F-actin. However, since degraded by protease in plasma and tissues, the plasma half-life of BK was short, the clinical role of BK was limited. RMP-7, the BK analog with a longer half-life, selectively increases the permeability of BTB, and has yet to be used clinically [9–11].

Histamine is formed by decarboxylation of histidine, and exists as an inactive conjugative form in mast cells and basophilic granulocytes, mainly in skin, bronchial mucosa, intestinal mucosa and nervous system. During an allergic response, these cells are activated to degranulate and result in histamine release, which in turn combines with histamine receptors and produces biological effects. In 1993, Nomura T et al. found that intracarotid administration of histamine increased regional cerebral blood flow in transplanted rat C6 glioma [12–14]. Administration of histamine H1 and H2 receptor antagonists, the former can still increase regional cerebral blood flow, while the latter could not, which implied increased regional cerebral blood flow by histamine may be associated with H2 receptors. Liu Y et al. found that B2 receptors mediated BK opening of BTB, the expression of B2 receptors in different glioma cells significantly differed from each other, and was correlated with BTB opening [15].

However, the expression of H2 receptors in different brain tumor tissues is unknown. In this study, we investigated the degree of H2-receptor-mediated permeability of BTB to histamine, as well as its potential mechanism.

## RESULTS

### Structure of endothelial cell and BTB after histamine treatment

**Figure 1:**
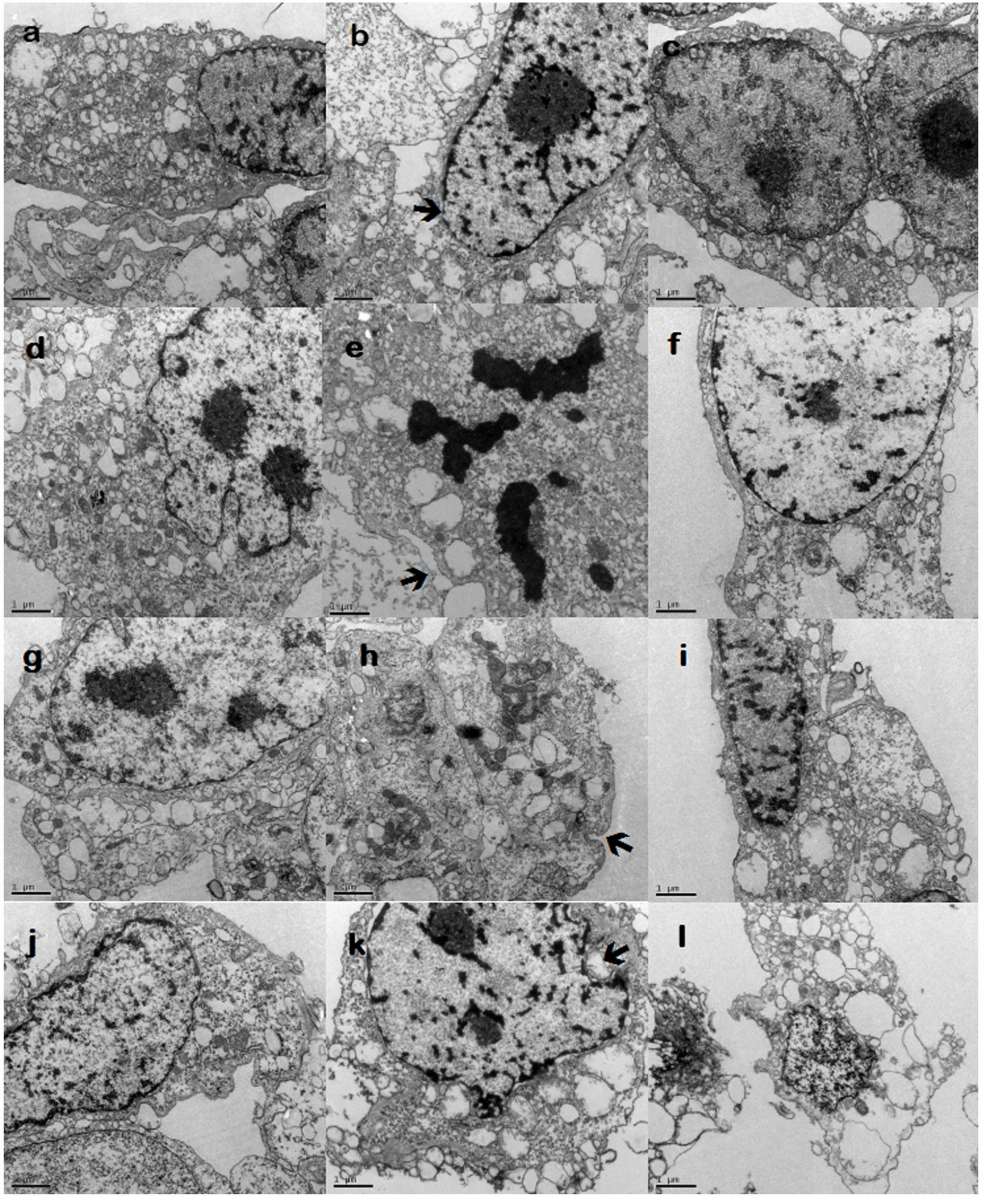
Transmission electron microscopy (TEM) analysis **a.** endothelial cells (blank group); **b.** endothelial cells (histamine group); **c.** endothelial cells (histamine + cimetidine group); **d.** endothelial cells + 9L (blank group); **e.** endothelial cells + 9L (histamine group); **f.** endothelial cells + 9L (histamine + cimetidine group); **g.** endothelial cells + C6 (blank group); **h.** endothelial cells + C6 (histamine group); **i.** endothelial cells + C6 (histamine+cimetidine group); **j.** endothelial cells + RG2 (blank group); **k.** endothelial cells + RG2 (histamine group); **l.** endothelial cells + RG2 (histamine + cimetidine group) Arrows show TJ; arrowheads show pinocytotic vesicles. Scale bar = 1 μm.

The cell structure of endothelial cells group was complete, with few pinosomes, and TJ appeared as a series of electron-dense zone (Figure 1a). The treatment of histamine damaged the integrity of mitochondria and cellular organelles. TJ displayed a clearly recognizable intercellular cleft between adjacent endothelial cells, and the number of pinosomes in the cytoplasm increased (Figure 1b). Whereas the treatment of cimetidine distorted the nuclear shape, and decreased the number of pinosomes in the cytoplasm (Figure 1c). The cell structure of endothelial and other tumor cell co-culture groups was incomplete. The cell nucleus was bigger, the mitochondria and the organelles were damaged. There were a few pinosomes in the cytoplasm. Similarly, after histamine treatment the mitochondria were swollen and damaged, and the structure of organelles was destroyed. Most of the cell structure was destroyed, disrupting the integrity of the tight cellular junctions and increasing the number of pinosomes in the cytoplasm. Cimetidine treatment distorted the nucleus, and decreased the number of pinosomes in the cytoplasm (Figure 1d–k).

### Effect of histamine on HRP permeability of endothelial cells and BTB

Histamine addition increased the HRP permeability of endothelial cells from day 1 to day 5, which was inhibited by cimetidine treatment. On day 1, compared with the control group of endothelial cells, the HRP permeability of the histamine group was significantly higher (P < 0.01). Though the HRP permeability decreased a little after cimetidine addition, its value was still higher than the control group (P < 0.01). The difference between histamine and cimetidine groups was also significant (P < 0.01). On days 3 and day 5, the difference between histamine and the control groups as well as between histamine and cimetidine groups was highly significant (P < 0.001). Similar phenomenon was observed in the RG2 cell+ endothelial cell group, 9L cell + endothelial cell group, and C6 cell + endothelial cell group groups.

**Figure 2:**
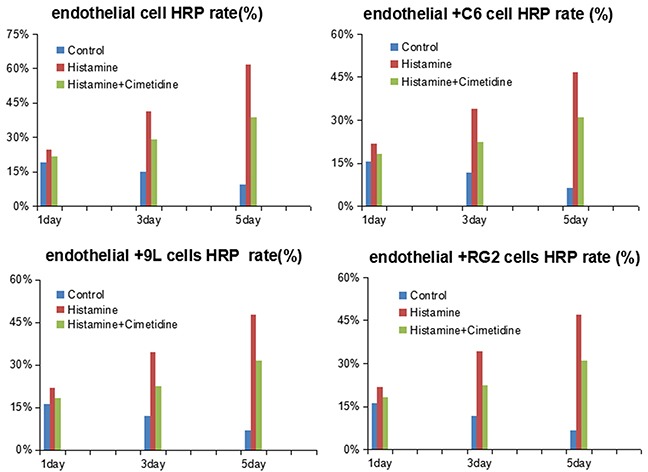
HRP rate of endothelial cells and BTB treated with histamine and cimetidine from day1 to day 5 Data represent means ± SD (n = 3 each).

However, when compared with the control group on day 1, the HRP permeability of the endothelial + C6 cell group as well as the other two BTB groups was not significant. Further, no significant difference was observed among the control, histamine and histamine + cimetidine groups on days 3 or day 5 (Figure 2).

### Difference of TEERs in endothelial cells and BTB

**Figure 3:**
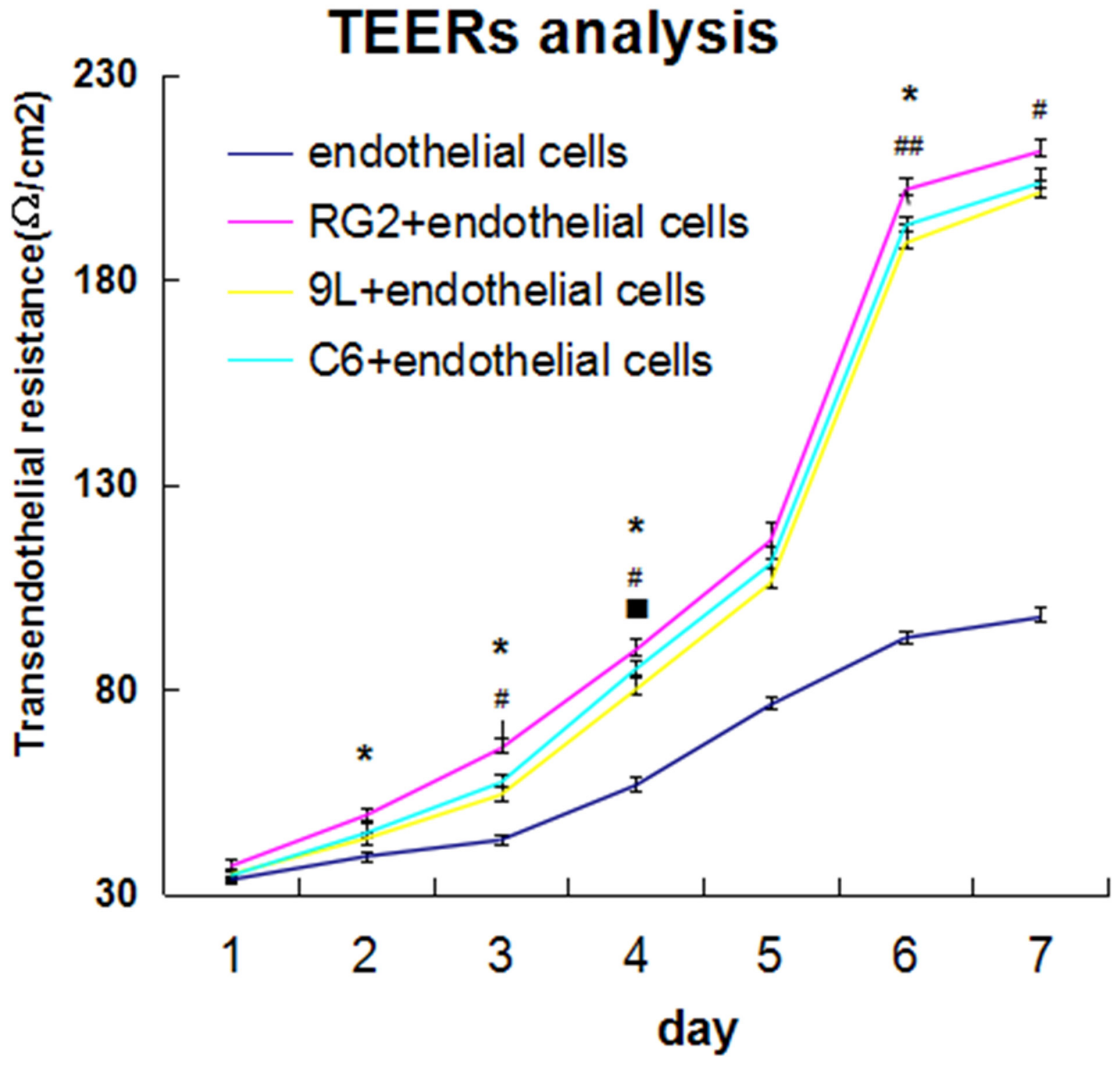
Transendothelial resistance detection (TEER) in endothelial cells and BTB from day 1 to day 7 TEER values in endothelial cells and BTB. endothelial cells(rose red curve), endothelial cell+RG2 (blue-green curve),endothelial cell+9L(yellow curve), endothelial cell+C6(dark blue curve), respectively. Data represent mean±SD (n=3, each). *P<0.05 9L versus RG2 group; #P<0.05; ##P<0.01 C6 versus RG2group; P<0.05 9Lversus C6 group.

TEER measurement was done using Millipore-ERS system. The results showed that brain capillary endothelial cell group TEERs differed little between days 1 and day 3, and increased from day 4 to day 6, and tended to be stable on day 7. In these three tumor co-cultured cell groups, TEERs differed little between days 1 and day 2, increased from day 3, increased rapidly from days 4 to day 6, and then plateaued on day 7. Of the tumor co-cultured cells, the TEER of RG2 cell + endothelial cell group was the highest, followed by C6 cell + endothelial cell group, and 9L cell + endothelial cell group. Further, compared with RG2 cell + endothelial cell group, the TEER values of 9L cell + endothelial cell group and C6 cell + endothelial cell group were significantly lower (P < 0.05). The significant difference between 9L cell + endothelial cell group and C6 cell + endothelial cell was also small, except on day 4 (Figure 3).

### Effect of histamine on TEERs of endothelial cells and BTB

**Figure 4:**
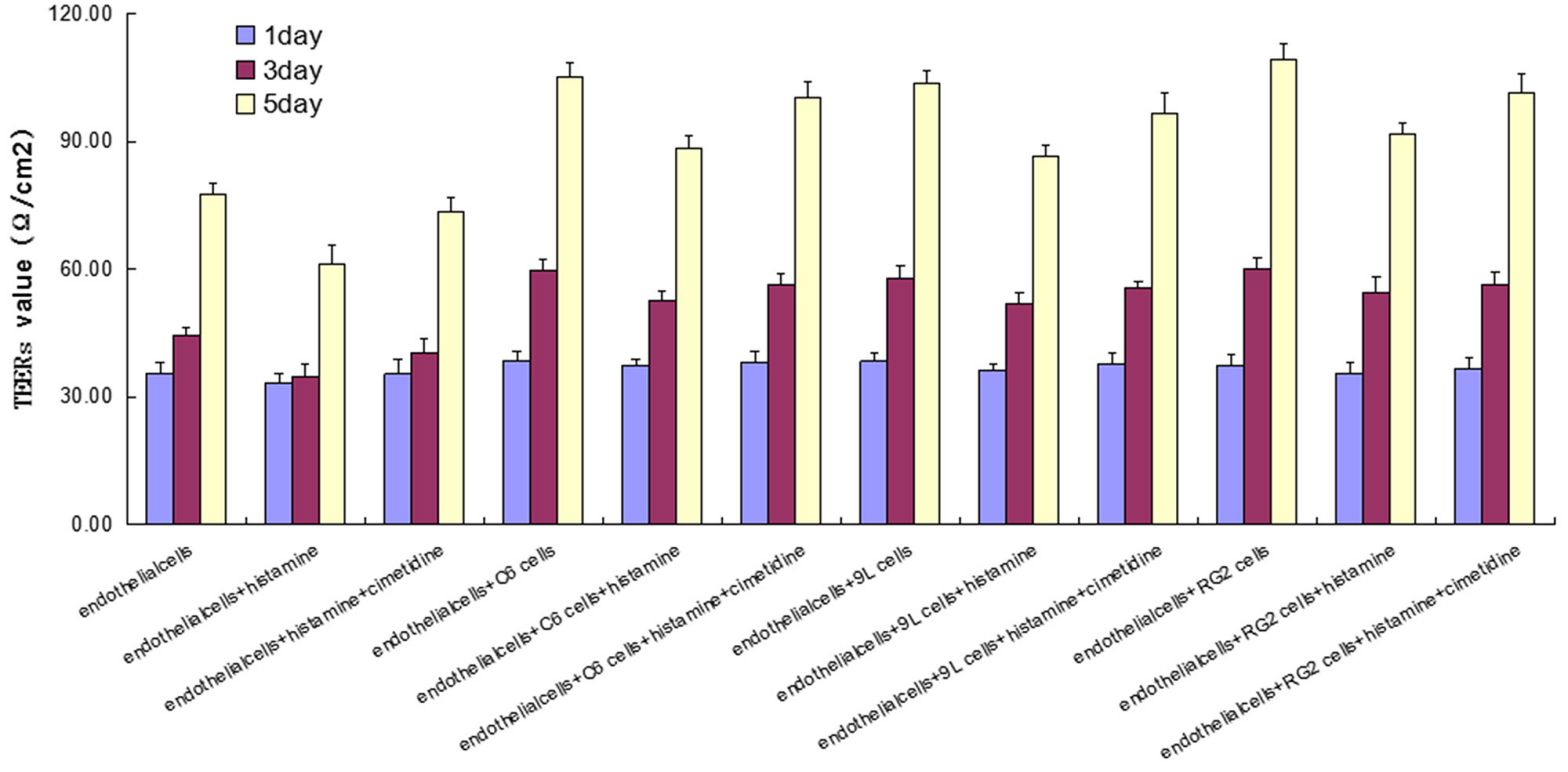
TEER in endothelial cell and other three endothelial cell and BTB treated with histamine and cimetidine from day 1 to day 5 Data represent means ± SD (n = 3 each)

The TEER value of brain capillary endothelial cell + histamine group was the lowest in all the endothelial cell groups. Compared with the endothelial cell day 1 group, the TEER value of endothelial cells + histamine day 1 group was significantly lower (P < 0.05). Compared with the endothelial cell day 3 group, the TEER value of endothelial cells + histamine day 3 group was significantly lower (P < 0.01). Compared with the endothelial cell day 5 group, the TEER value of endothelial cells + histamine day 5 group was also significantly lower (P < 0.05). Further, compared with the endothelial cells and histamine + cimetidine group, the endothelial cells and histamine group had lower TEER value on day 3 (P < 0.05) and day 5 (P < 0.01). Similar phenomena were also observed in the other three cell lines (RG2, 9L, C6 cells) (Figure 4).

### H2 receptor mRNA expression in endothelial cells and BTB

**Figure 5:**
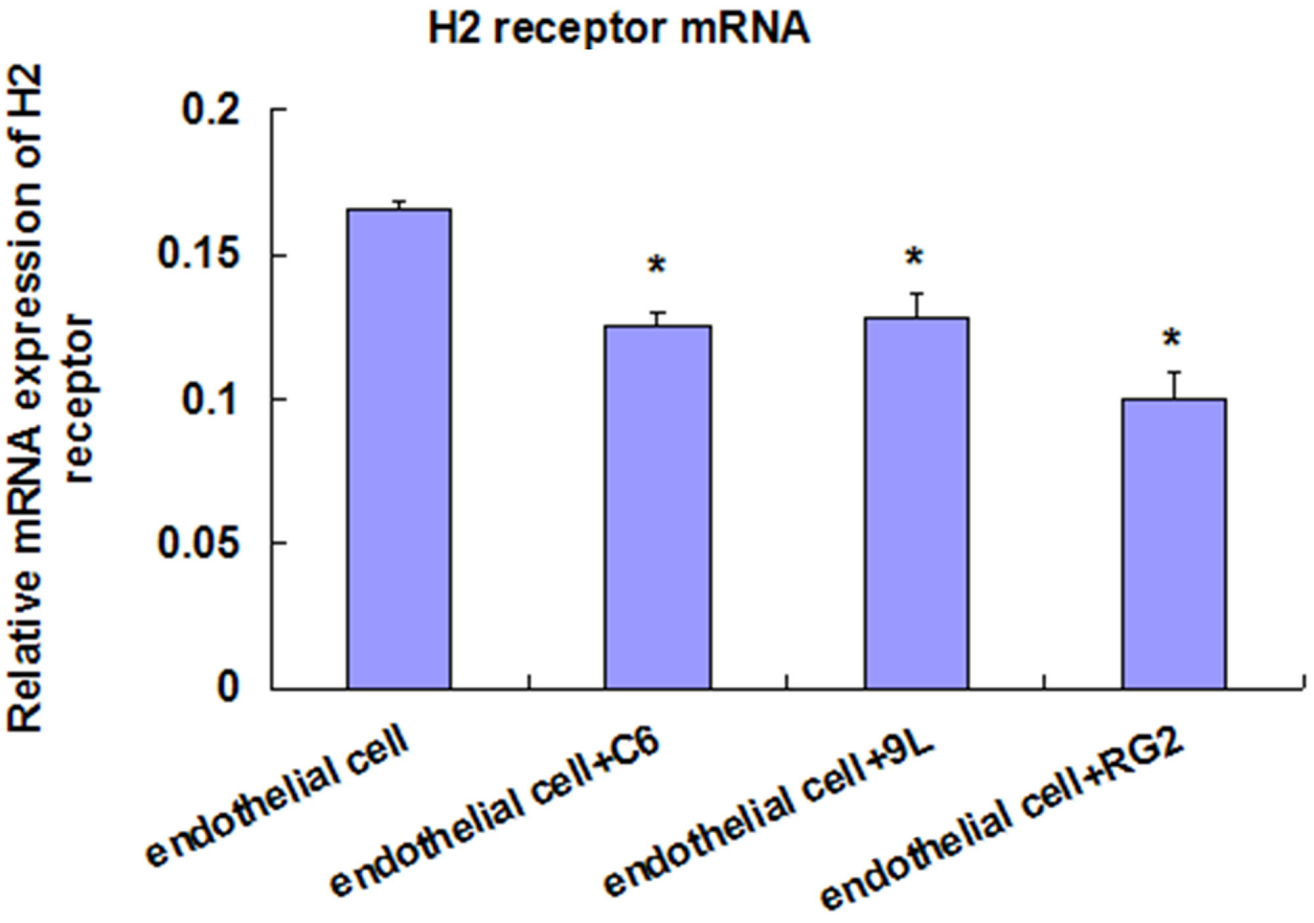
Real-Time PCR of H2 receptor mRNA detection in endothelial cell and BTB Data represent mean±SD (n=3, each). *P<0.05 *** P<0.001 versus endothelial cell.

Compared with the control group (endothelial cells), the H2 receptor mRNA of endothelial cells + C6 group, endothelial cells + 9L group, and endothelial cells + RG2 group were all lower, and significantly different (P < 0.05). The expression of H2 receptor in RG2 BTB was the highest, and was the lowest in C6 BTB. However, the H2 receptor mRNA level in the three groups (RG2, 9L, and C6 cells) was not significantly different (Figure 5).

### Expression of caveolin-1, ZO-1, occludin, claudin-5 and H2R in endothelial cells and BTB

**Figure 6:**
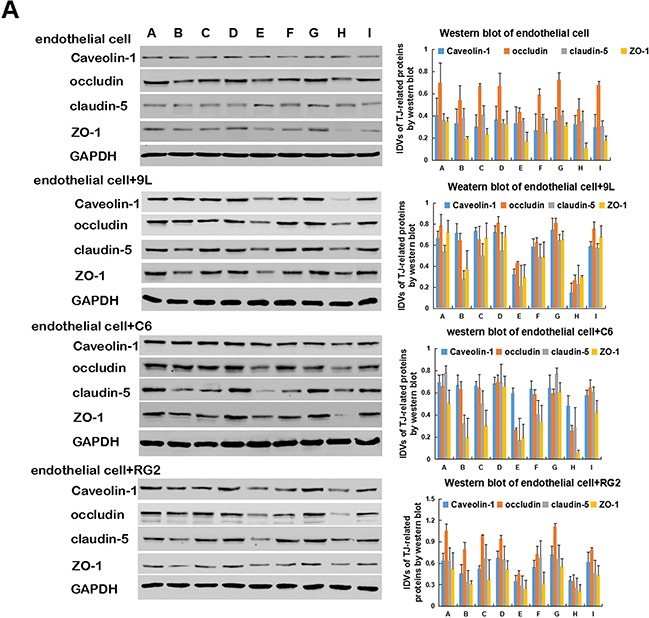

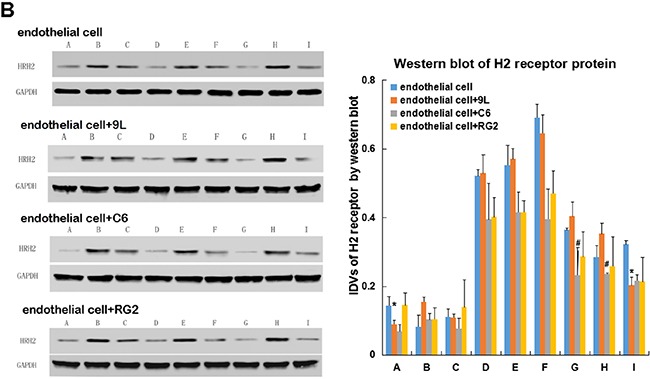
Western blot of TJ-related proteins, caveolin-1 and H2R expression in endothelial cell and BTB **A.** Representative protein expression and their integrated light density values (IDVs) of TJ-related protein (ZO-1, occludin, claudin-5) and caveolin-1 expression levels in day 1, day 3 and day 5 in endothelial cell and BTB are shown, using GAPDH as an endogenous control. **B.** H2R expression levels in day 1, day 3 and day 5 in endothelial cell and BTB, using GAPDH as an endogenous control. Representative protein expression and their integrated light density values (IDVs) of TJ-related protein, caveolin-1 and H2R in endothelial cell and BTB are shown. Data represent mean ±SD (n=3, each). *P<0.05 versus endothelial cell. # P<0.05 versus endothelial cell+C6 Groups: Day 1 A. Control B. Histamine C. Histamine + Cimetidine Day 3 D. Control E. Histamine F. Histamine + Cimetidine Day 5 G. Control H. Histamine I. Histamine + Cimetidine.

Histamine significantly decreased the protein levels of ZO-1, occludin, and claudin-5 in endothelial and tumor cells. Though the expression of TJ-related proteins among the three groups (RG2, 9L, C6 cells) was significantly different, it was not consistent. In addition, histamine treatment decreased the expression of caveolin-1 (Figure 6A).

Compared with the control group (endothelial cells) on day 3 and 5, the H2 receptor protein of endothelial cells + C6 group and endothelial cells + RG2 group were lower (P>0.05), and the H2 receptor protein of endothelial cells + 9L group was similar (P>0.05) (Figure 6B).

### Immunofluoresence localization of ZO-1 in endothelial cells and BTB

**Figure 7:**
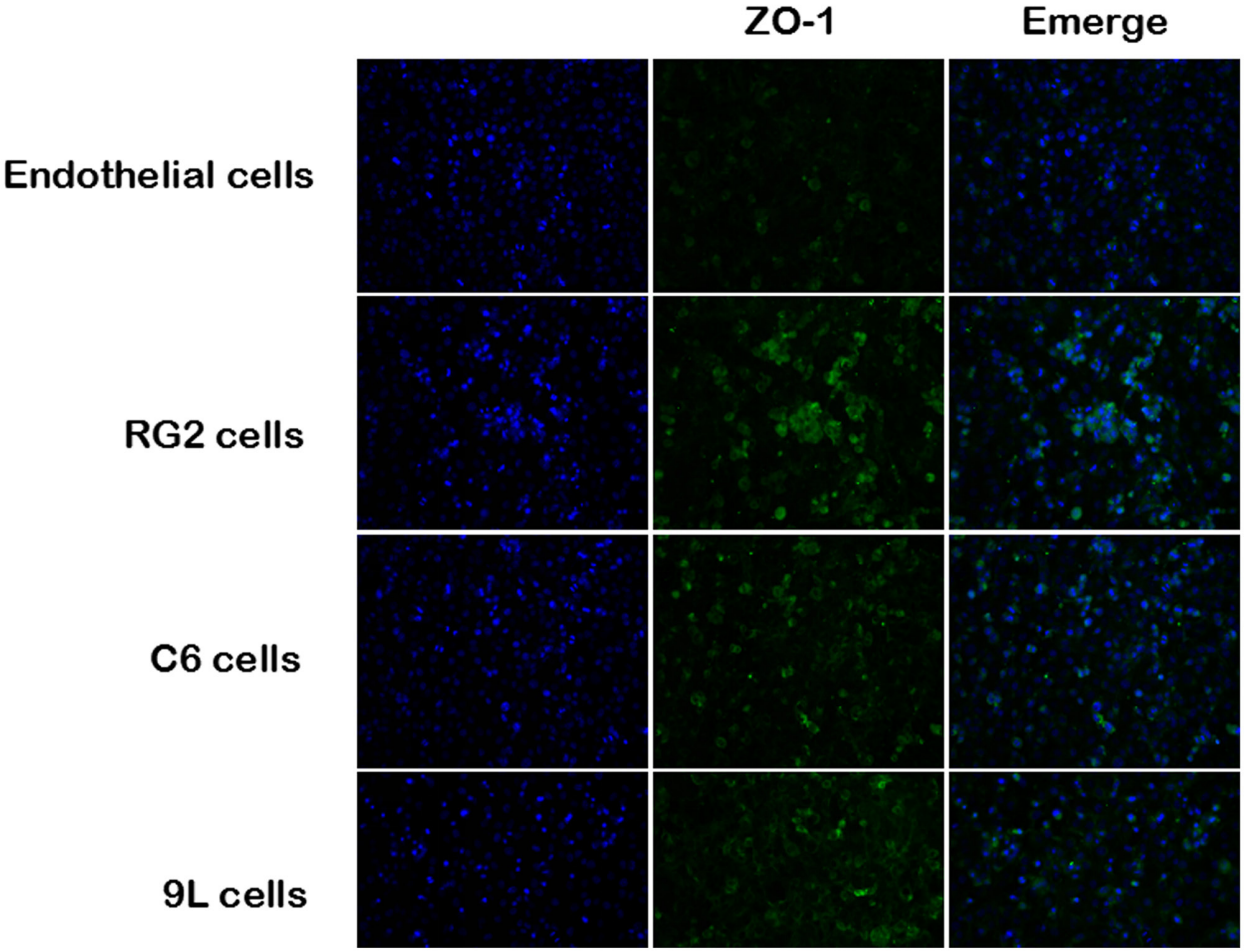
Immunofluoresent localization of ZO-1 in endothelial cell and BTB Immunofluorescence staining of ZO-1 in endothelial cell and BTB. ZO-1 (green) labeled with secondary antibody against anti-ZO-1 antibody; nuclei (blue) are labeled with DAPI. Images are representative of 3 independent experiments. Original magnification:100×. Scale bar = 50μm.

Immunofluorescence method was conducted to determine the expression and distribution of ZO-1, in endothelial and other three endothelial cell and tumor cell co-culture groups. Nuclei counterstained with DAPI are shown in blue. The expression of ZO-1 is shown in green (Figure 7).

**Figure 8:**
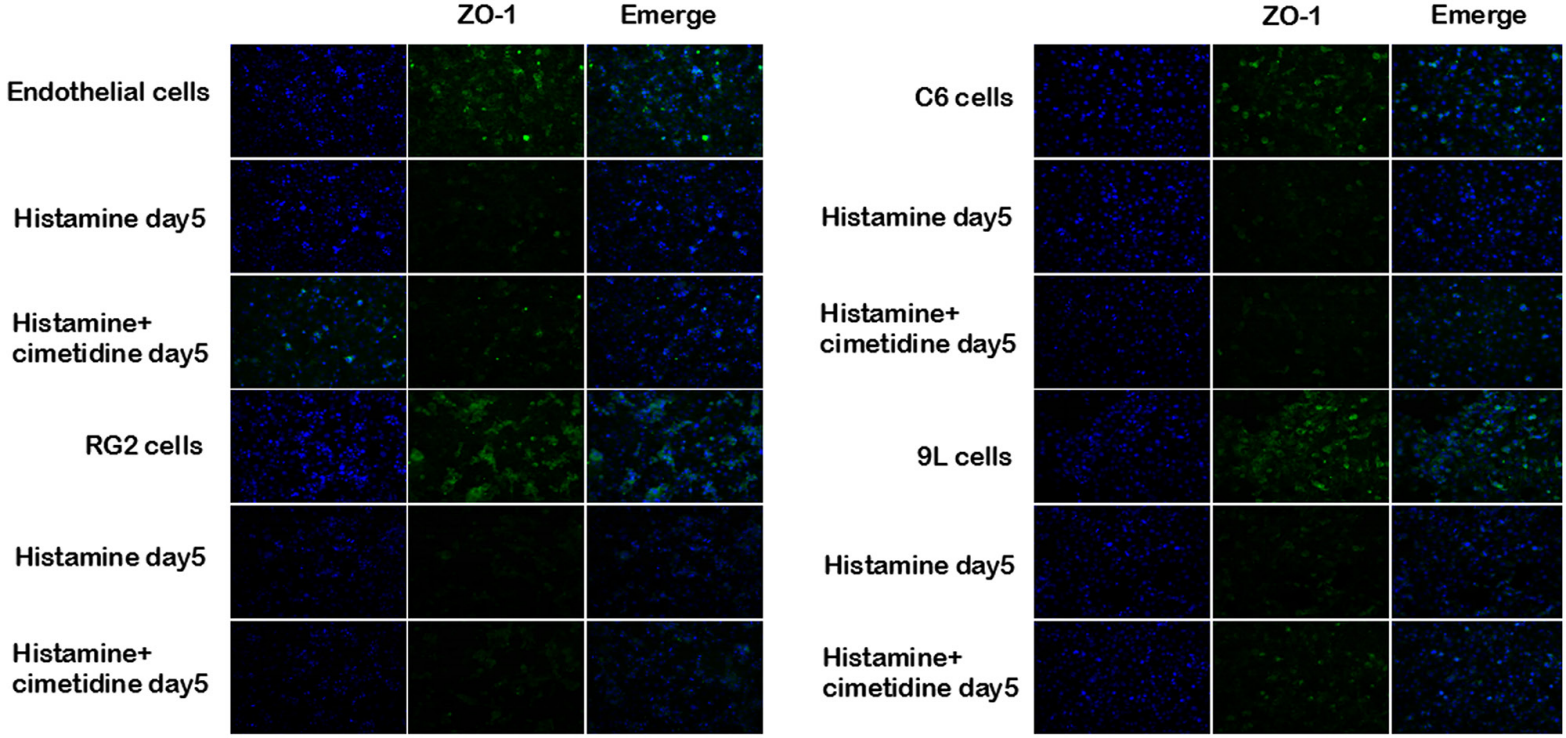
Immunofluoresent localization of ZO-1 in endothelial cells and BTB after treatment with histamine and cimetidine Immunofluorescence staining of ZO-1 in endothelial cell and BTB treated with histamine and cimetidine from day1 to day 5. ZO-1 (green) labeled with secondary antibody against anti-ZO-1 antibody; nuclei (blue) are labeled with DAPI. Images are representative of 3 independent experiments. Original magnification:100×. Scale bar = 50μm.

The distribution of ZO-1was observed not only in endochylema, cell membrane, but also in cellular tight junctions. ZO-1 showed a continuous distribution along the cell border of the endothelial cells in the control group and discontinuously distributed in the tumor co-cultured cell group. After histamine treatment, the expression of ZO-1 turned to weak, and this effect was partially reversed by cimetidine in the BTBs (Figure 8).

## DISCUSSION

In this study, we observed that histamine increased the permeability of the BTB, via downregulation of the TJ-associated proteins ZO-1, occludin, and caludin-5, whereas the effect could be inhibited by H2 receptor blocker cimetidine. More, the fact that the degree of H2 receptor mRNA expression was correlated to the extent of histamine opening in BTB suggested that the expression of H2 receptor might predict histamine-mediated BTB permeability.

BTB contains an efficient cluster of TJs, which significantly reduce drug permeability. To identify the role of histamine-mediated permeability of BTB, an *in vitro* BTB model was established. The integrity and permeability of BTB were evaluated by TEER and HRP permeability assays, respectively. The results showed that histamine reduced the basal TEER value, and increased the HRP rate of endothelial cells and the other three BTBs. This effect was inhibited by cimetidine. Further, TEM analysis also showed that histamine destroyed the integrity of TJ and increased the number of pinosomes in the cytoplasm, which suggested its role in opening BTB.

In addition, we found that H2 receptor mRNA expression was highest in RG2 BTB, and the lowest in C6 BTB. The expression of H2 receptor mRNA was inversely correlated with the TEER value, which indicated that the expression of H2 receptor might predict histamine-induced BTB permeability. These results are consistent with the conclusion of Nomura T [12]. He reported that intracarotid administration of histamine together with Azo-Blue was found in glioma and glioma tissues, but not in normal brain tissue, indicating that the effect of histamine-induced increase in BTB permeability or altered specificity of receptors. Inamura T [13] also demonstrated that intracarotid histamine infusion increased BTB permeability, via H2 receptors.

However, the potential molecular mechanisms underlying regulation of BTB function by histamine were still unclear. Further, we investigated whether histamine suppressed the expression levels of TJ-related proteins and caveolin-1 in BTB model. ZO-1, occludin and claudin-5 are important components of TJs. ZO-1 is a member of the membrane-associated guanylate kinase-like protein family, located on a cytoplasmic membrane surface. Occludin is an integral plasma membrane protein. Claudin-5 regulates the function of the tight junctions [16–18]. The treatment of histamine resulted in a significant downregulation of the protein levels of ZO-1, occludin, and claudin-5, as evidenced by Western blot assays. In addition, the immunofluorescence result of ZO-1 was consistent with the western blot study.

In summary, the present study revealed that histamine opened BTB by down-regulating the TJ-associated proteins. The levels of H2 receptor expression was correlated with the histamine-induced BTB permeability. Based on these findings, histamine and its receptor should be paid more attention in tumor therapies in future.

## MATERIALS AND METHODS

### Cell culture and *in vitro* BTB models

**Figure 9:**
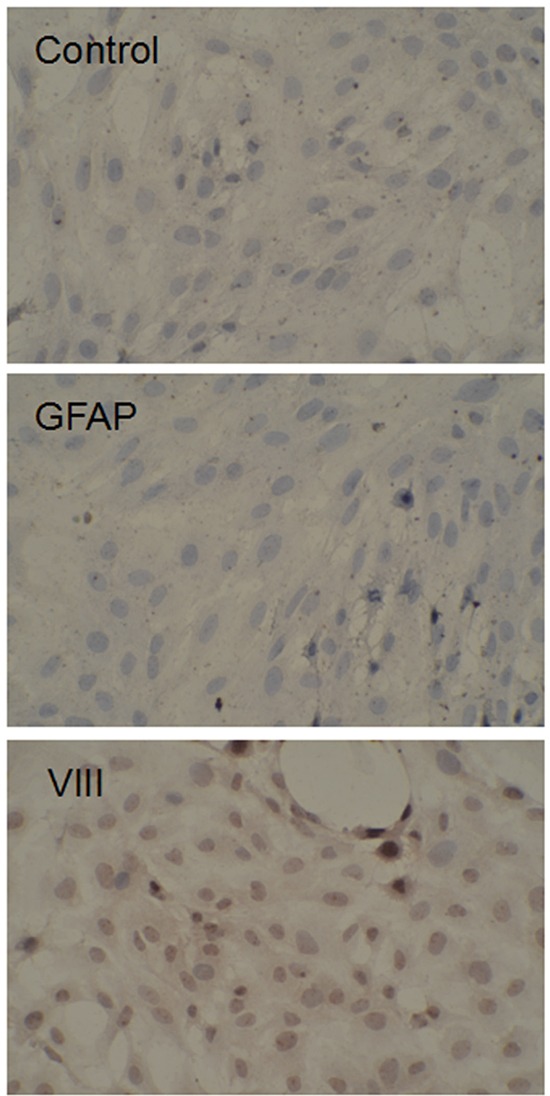
Expression of VIII and GFAP VIII and GFAP expression were detected by immunohistochemical assay immunohistochemical staining of VIII and GFAP in endothelial cell and BTB VIII and GFAP (brown) labeled with secondary antibody against anti-VIII and anti anti-GFAP antibody; nuclei (blue) are labeled with DAPI. Images are representative of 3 independent experiments. Original magnification: 200×.

The brain microvascular endothelial cells of 2-week-old Sprague-Dawley (SD) rats (provided by Shanghai Lab. Animal Research Center) were separated and cultured in high glucose DMEM (20% FBS, 1 mg/mL heparin, 1ng/mL β-FGF, 10 ng/mL VEGF, 100U/mL penicillin, 100 μg/mL streptomycin). In order to determine the separation in the brain microvascular endothelial cells, the relative expression of markers VIII and GFAP was measured by immunohistochemical assay (Figure 9).

The RG2 glioma, 9L, and C6 cell lines were also maintained in DMEM medium with 10% calf serum. All cells were cultured at 37°C in a humidified atmosphere containing 5% CO_2_. Cells at passages 30 to 40 were used for the following experiments.

To establish in vitro BTB models, 1 × 10^6^ C6 cells inoculated in a 10-cm diameter culture dish, and 5 × 10^5^ endothelial cells were inoculated on a 12-well plate cell climbing film. After 12-h cultivation, the 12-well-plate cell climbing film was covered with endothelial cells into the C6 cell inoculated culture dish and co-cultured similar to other co-culture groups.

### Transmission electron microscopy (TEM)

According to the standard procedures, semi-thin and ultrathin sections were obtained and stained with uranyl acetate and lead citrate. The changes in TJ and pinosomes were examined by TEM (JEM-1230EX; n=5, each).

### Horseradish peroxidase (HRP) permeability

The experiment was divided into 4 groups: brain capillary endothelial cells (control group), RG2 cell + endothelial cell co-culture group, 9L cell + endothelial cell co-culture group, and C6 cell + endothelial cell co-culture group. Cells in different groups were trypsinized, and seeded in transwells, to ensure adaptation to complete medium. Cells were incubated at 37°C, in 5% CO_2_.

HRP permeability was measured. HRP was dissolved in DMEM culture solution, supplemented with 650 μL of 0.002mg/mL HRP-DMEM in donor transwell and 850 μL of DMEM in receptor transwell. The liquid level inside and outside was consistent to minimize the effect of differential pressure on permeability. A 50 μL sample was obtained from donor and receptor transwells on days 1, 3 and 5, respectively, transferred to the enzyme label plate and stored at 4°C. After sampling, the substrates were added and the color development time was 15 min. After reaction termination, the absorbance of donor and receptor transwells was determined at 450 nm. The HRP permeability was calculated using the equation P_HRP_% = C_HRP_ donor transwell × V_HRP_ receptor transwell/C_HRP_ donor transwell × V_HRP_ donor transwell. (C represents concentration, V represents volume)

### Transendothelial resistance (TEER) detection

TEER was performed according to protocols recommended by Millipore Company, MA, USA. The experiment was divided into 4 groups : brain capillary endothelial cell group, RG2 cell + endothelial cell co-culture group, 9L cell + endothelial cell co-culture group, and C6 cell + endothelial cell co-culture group. Cells in different groups were trypsinized, seeded in transwells, and transferred to new complete medium.

### Experiment 1

Cells were cultured and measured for 7 consecutive days at 37°C incubator, in 5% CO_2_. The culture solution was completely abandoned before each measurement and rinsed with PBS 3 times, and transferred to transwells in PBS. The resistance inside and outside the transwell was determined using the resistance instrument (Millipore Company, Boston, USA). The value was then subtracted from the resistance of the control group and divided by membrane area of transwell, to obtain the TEER values (Ω/cm^2^).

### Experiment 2

Cells were cultured and measured for 5 consecutive days after incubation at 37°C and 5% CO_2_. Each cell line comprised three groups: control, histamine-treated, and histamine followed by cimetidinetreated groups. The rest of the experiment procedure was similar to that of experiment 1.

### Real-time PCR

Total RNA was extracted using TRIzol reagent following the manufacturer's protocol (TakaRa Biotechnology Co., Ltd, Dalian, China). Reverse transcription was performed using the high-capacity cDNA reverse transcription kits (Fermentas, Lithuania) according to the manufacturer's instruction. Real-time PCR was performed using the ABI PRISM 7300 Sequence Detection System (Applied Biosystems, Bedford, MA) and SYBR Green Reagent Kit (Thermo, Waltham, USA) to determine the mRNA expression. The reaction conditions were as follows: 37°C, for 60 min, 85°C, for 5 min, 4°C, for 5 min and stored at −20°C. All quantitative RT-PCR analyses were conducted using ABI Prism 7300 SDS Software. H2R: Forward: 5′; GAACAGCAGGAACGAGAC 3′;, Reverse : 5′; AGTAGCGGGAGGTAGAAG 3′;;

GAPDH: Forward: 5′; CACCCACTCCTCCACCTT TG 3′;, Reverse: 5′; CCACCACCCTGTTGCTGTAG 3′;.

### Western blot

Cells were removed from the incubator and rinsed with pre-cooled 1×PBS twice, followed by addition of lysate with protease and phosphatase inhibitors. Cells were lysed at 4°C. The cells were transferred to 1.5 mL EP tube, heated above 95°C for 10 min, and centrifuged at 4°C, and 12000g for 10 min. After determination of protein levels, it was stored at −80°C. The protein bands were separated by SDS-PAGE, and electrophoretically transferred to a transmembrane, blocked with appropriate antibodies to occludin, claudin-5, caveolin-1, H2R(Abcam, UK) and ZO-1(Santa Cruz, CA, USA), followed by incubation and coloration.

### Immunofluorescence

Immunofluoresent localization of ZO-1 in endothelial cell and BTB was studied. More, after treatment by histamine as well as cimetidine, immunofluoresent localization of ZO-1 in endothelial cells and BTB was also investigated. All cells in the different groups were fixed with 4% formaldehyde for 10 min at room temperature and blocked with PBS containing 10% goat serum, 0.3 M glycine, 1% BSA and 0.1% Tween for 2h at room temperature. Staining of the treated cells with ab59720 (0.5 μg/mL) was performed overnight at 4°C in PBS containing 1% BSA and 0.1% Tween. Nuclei were counterstained with DAPI and are shown in blue. The primary antibody was ZO-1(Santa Cruz, CA, USA).

### Statistical analysis

All statistical analyses were carried out on an intention-to-treat basis using the SPSS 15.0 software package with statistical significance which was assigned with P-values of <0.05.
